# Supplementary material for: Clinical Characteristics and Follow-Up of Children with Primary Haematogenous Osteomyelitis and Septic Arthritis: Eight Years of Experience from Hungary
Source: Antibiotics (Basel). 2025 Aug 11;14(8):821. doi: 10.3390/antibiotics14080821 (PMC12382815; doi:10.3390/antibiotics14080821)
Supplement: Supplementary file 1 [file antibiotics-14-00821-s001.zip › antibiotics-3774621-supplementary.pdf]

Supplementary file S1.

Disease / imaging modality	X-ray	Ultrasound	Magnetic resonance imaging
AHO<br>*(minimum of 1)*	May show soft tissue swelling<br><br>Often normal in early phase<br><br>Subtle changes may take 7-10 days to appear:<br>- Regional osteopenia<br>- Periosteal reaction/thickening (periostitis)<br>- Focal bony lysis or cortical loss<br>- Endosteal scalloping<br>- Loss of trabecular bone architecture<br>- New bone apposition<br>- Peripheral sclerosis	Unable to visualize within the bone<br><br>Rarely:<br>- Soft tissue swelling<br>- Periosteal elevation<br>- Subperiosteal abscess	Recommended modality for suspected AHO = most sensitive for early disease<br><br>Early phase:<br>- Bone marrow oedema (decreased T1 with increased T2/STIR signal)<br>- Subperiosteal/intramedullary abscess<br>- Soft tissue involvement<br>- Loss of normal marrow fat<br>- Cortical destruction<br><br>Concordant low signal T1 and high signal on fluid-sensitive sequences<br>T1: intermediate to low signal central component surrounding bone marrow T2: bone marrow high signal<br>T1+gadolinium contrast:<br>- Post-contrast enhancement of bone marrow, abscess margins, periosteum
SA<br>*(minimum of 1)*	Early phase: often negative.<br>- Joint space widening<br>- Joint space narrowing = cartilage destruction<br>- Displacement of joint structures<br>- Soft tissue swelling<br><br>Late phase:<br>- Destruction of the subchondral bone	Early phase:<br>- Joint effusion<br>- Synovial membrane thickening<br>- Increased perisynovial vascularity	MRI is sensitive/specific for early cartilaginous damage with effusion.<br>- Synovial enhancement and thickening<br>- Joint effusion<br>- Bone marrow signal change if adjacent osteomyelitis<br>- Perisynovial edema<br><br>T1: low signal within the subchondral bone<br>T2: thin rim of subchondral edema; pericapsular edema.<br>T1+gadolinium contrast: synovial and pericapsular enhancement

Characteristics of X-ray, US and MRI imaging in paediatric primary haematogenous osteomyelitis and septic arthritis

REFERENCES

Haas, J.P. Osteomyelitis, septische Arthritis, nichtbakterielle Osteomyelitis, SAPHO-Syndrom bei Kindern: Was möchte der Kliniker vom Radiologen wissen? [Osteomyelitis, septic arthritis, nonbacterial osteomyelitis, SAPHO syndrome in children : What clinicians want to know from the radiologist?]. *Radiologie (Heidelb)* **2025**. German.

Uhl, M.; Hufnagel, M. Hämatogene Osteomyelitis im Kindesalter: Ein gefürchteter pädiatrischer Notfall [Hematogenous osteomyelitis in children : A feared pediatric emergency]. *Radiologie (Heidelb)* **2025**. German.

Hospach, T.; Kallinich, T.; Martin, L.; V Kalle, T.; Reichert, F.; Girschick, H.J.; Hedrich, C.M. Arthritis und Osteomyelitis im Kindes- und Jugendalter – bakteriell und nichtbakteriell [Arthritis and osteomyelitis in childhood and adolescence-Bacterial and nonbacterial]. Z Rheumatol **2025**, *84*(4):276-287. German.

Hunter, S.; Brown, E.; Crawford, H.; Grant, C. Optimal Timing for Advanced Imaging in Childhood Bone and Joint Infection. *J Pediatr Orthop* **2025**, *45*(2):e166-e171.

Jaramillo, D.; Dormans, J.P.; Delgado, J.; Laor, T.; St Geme, J.W. 3rd. Hematogenous Osteomyelitis in Infants and Children: Imaging of a Changing Disease. *Radiology* **2017**, *283*(3):629-643.

Supplementary file S2.

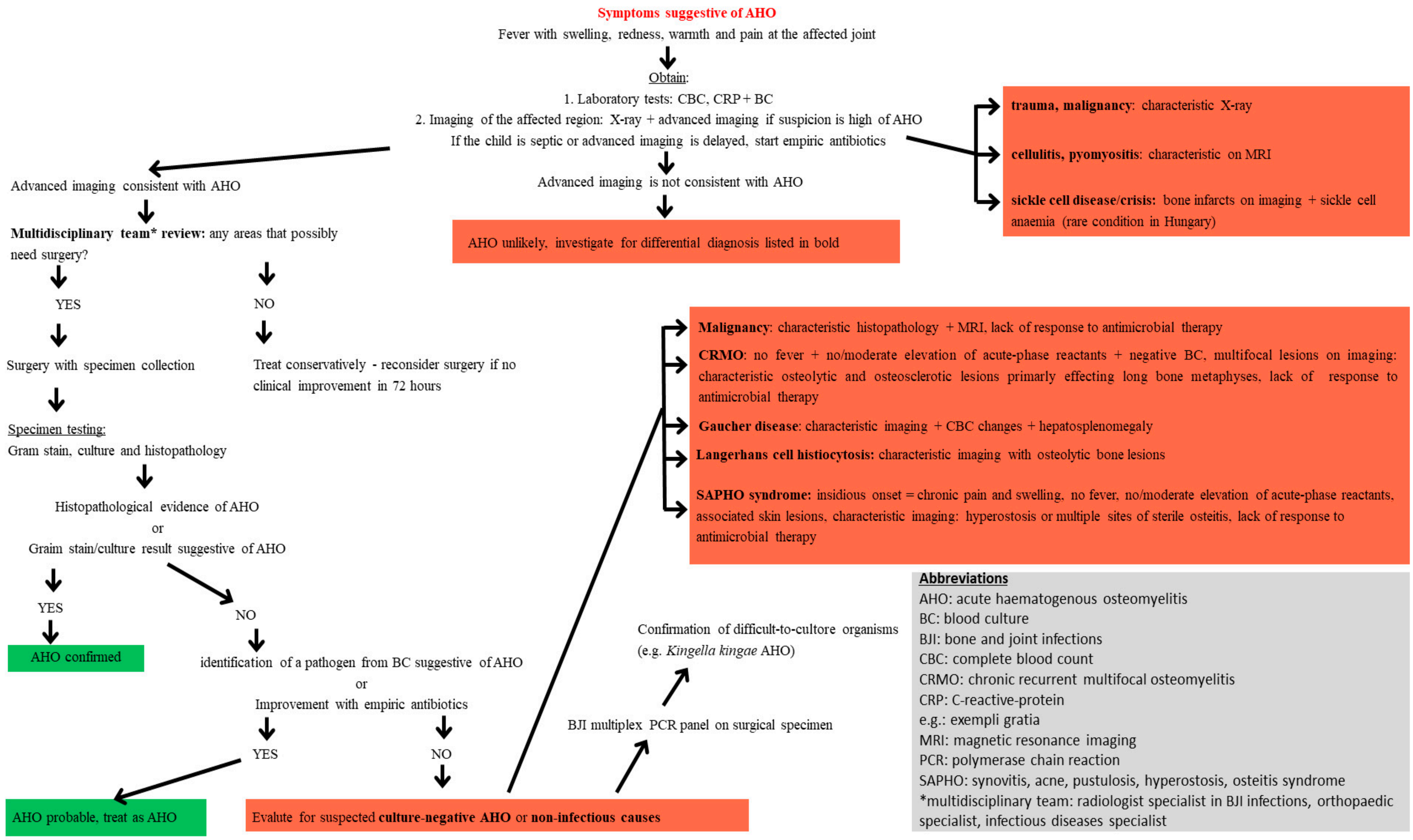

Differential diagnostic algorithm for acute haematogenous osteomyelitis (AHO) in children. Differential diagnoses are listed in bold.

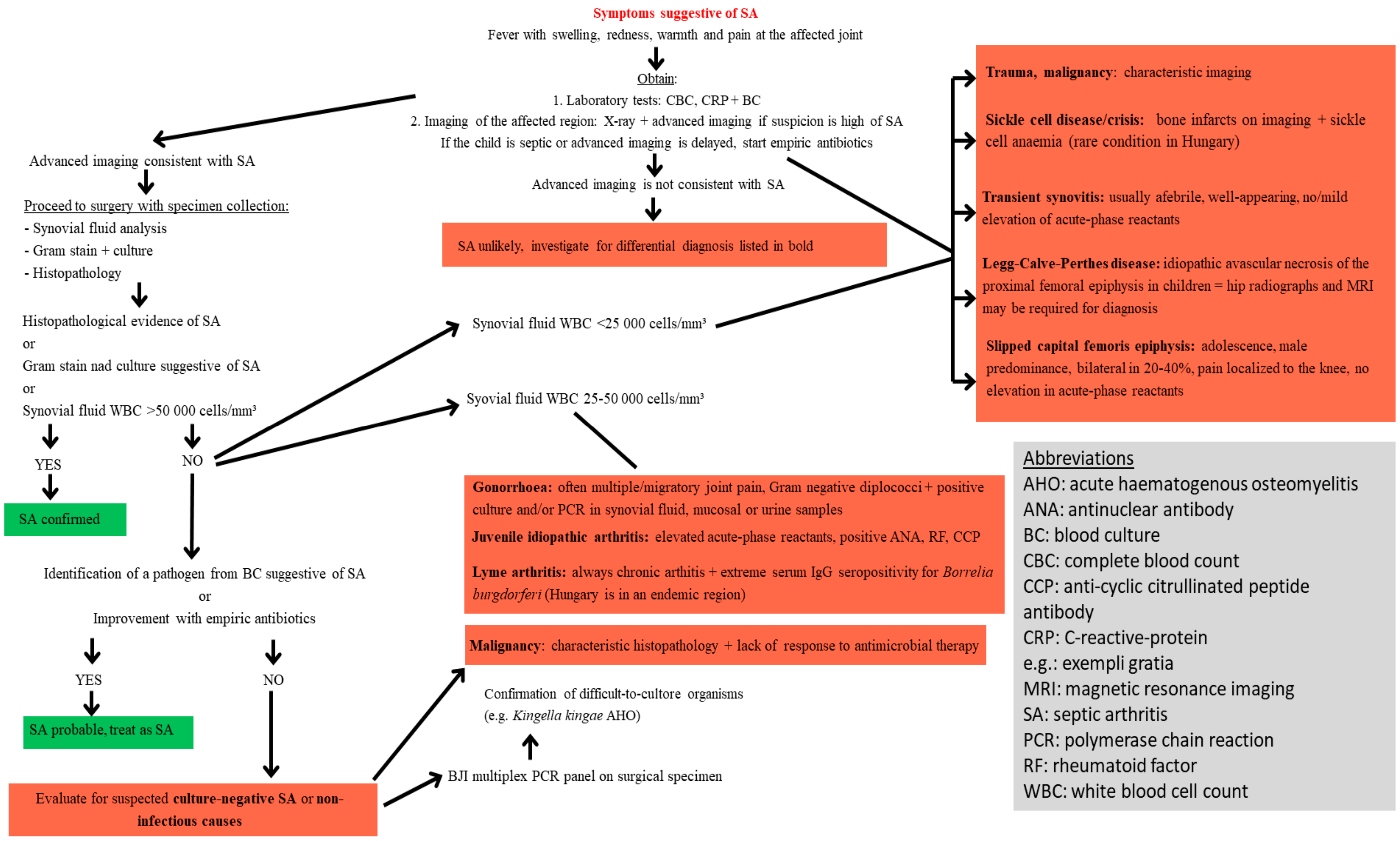

Differential diagnostic algorithm for septic arthritis (SA) in children. Differential diagnoses are listed in bold.

REFERENCES

Woods, C.R.; Bradley, J.S.; Chatterjee, A.; Copley, L.A.; Robinson, J.; Kronman, M.P.; Arrieta, A.; Fowler, S.L.; Harrison, C.; Carrillo-Marquez, M.A.; Arnold, S.R.; Eppes, S.C.; Stadler, L.P.; Allen, C.H.; Mazur, L.J.; Creech, C.B.; Shah, S.S.; Zaoutis, T.;, Feldman, D.S.; Lavergne, V.. Clinical Practice Guideline by the Pediatric Infectious Diseases Society and the Infectious Diseases Society of America: 2021 Guideline on Diagnosis and Management of Acute Hematogenous Osteomyelitis in Pediatrics. *J Pediatric Infect Dis Soc* **2021**; *10*(8):801-844.

Woods, C.R.; Bradley, J.S.; Chatterjee, A.; Kronman, M.P.; Arnold, S.R.; Robinson, J.; Copley, L.A.; Arrieta, A.C.; Fowler, S.L.; Harrison, C.; et al. Clinical Practice Guideline by the Pediatric Infectious Diseases Society (PIDS) and the Infectious Diseases Society of America (IDSA): 2023 Guideline on Diagnosis and Management of Acute Bacterial Arthritis in Pediatrics. *J Pediatric Infect Dis Soc* **2024**, *13*, 1–59.

Saavedra-Lozano, J.; Falup-Pecurariu, O.; Faust, S.N. et al. Bone and Joint Infections. *The Pediatric Infectious Disease Journal* **2017**, *36*, 788.

Dartnell, J.; Ramachandran, M.; Katchburian, M. Haematogenous acute and subacute paediatric osteomyelitis: a systematic review of the literature. *J Bone Joint Surg Br.* **2012**, *94*(5):584-95.

Cochard, B.; De Marco, G.; Bazin, L.; Vazquez, O.; Di Laura Frattura, G.; Steiger, C.N.; Dayer. R.; Ceroni, D. Biological Predictors of Osteoarticular Infection Due to *K. kingae*-A Retrospective Cohort Study of 247 Cases. *Microorganisms* **2023**, *11*(9):2130.

Zhorne, D.J.; Altobelli, M.E.; Cruz, A.T. Impact of antibiotic pretreatment on bone biopsy yield for children with acute hematogenous osteomyelitis. *Hosp Pediatr* **2015**, *5*(6):337-41.

Jaramillo, D.; Dormans, J.P.; Delgado, J.; Laor, T.; St Geme, J.W. 3rd. Hematogenous Osteomyelitis in Infants and Children: Imaging of a Changing Disease. *Radiology* **2017**, *283*(3):629-643.

Landin, L.A.; Danielsson, L.G.; Wattsgård, C. Transient synovitis of the hip. Its incidence, epidemiology and relation to Perthes' disease. *J Bone Joint Surg Br* **1987**, *69*(2):238-42.

Laine, J.C.; Novotny, S.A.; Tis, J.E.; Sankar, W.N.; Martin, B.D.; Kelly, D.M.; Gilbert, S.R.; Shah, H.; Joseph, B.; Kim, H.K.W.; International Perthes Study Group. Demographics and Clinical Presentation of Early-Stage Legg-Calvé-Perthes Disease: A Prospective, Multicenter, International Study. *J Am Acad Orthop Surg* **2021**, *29*(2):e85-e91.

Herngren, B.; Stenmarker, M.; Vavruch, L.; Hagglund, G. Slipped capital femoral epiphysis: a population-based study. *BMC Musculoskelet Disord* **2017**, *18*(1):304.

Jones, O.Y.; Spencer, C.H.; Bowyer, S.L.; Dent, P.B.; Gottlieb, B.S.; Rabinovich, C.E. A multicenter case-control study on predictive factors distinguishing childhood leukemia from juvenile rheumatoid arthritis. *Pediatrics* **2006**, *117*(5):e840-4.

Schnabel, A.; Range, U.; Hahn, G.; Siepmann, T.; Berner, R.; Hedrich, C.M. Unexpectedly high incidences of chronic non-bacterial as compared to bacterial osteomyelitis in children. *Rheumatol Int* **2016**, *36*(12):1737-1745.
